# Skeletal muscle is associated with exercise tolerance evaluated by cardiopulmonary exercise testing in Japanese patients with chronic obstructive pulmonary disease

**DOI:** 10.1038/s41598-021-95413-9

**Published:** 2021-08-05

**Authors:** Hiroki Tashiro, Koichiro Takahashi, Masahide Tanaka, Hironori Sadamatsu, Yuki Kurihara, Ryo Tajiri, Ayako Takamori, Hiroyuki Naotsuka, Hiroki Imaizumi, Shinya Kimura, Naoko Sueoka-Aragane

**Affiliations:** 1 Division of Hematology, Respiratory Medicine and Oncology, Department of Internal Medicine, Faculty of Medicine, Saga University, 5-1-1 Nabeshima, Saga, Saga Prefecture 849-8501 Japan; 2 Clinical Research Center, Saga University Hospital, Saga, Japan; 3 Advanced Comprehensive Functional Recovery Center, Saga University Hospital, Saga, Japan

**Keywords:** Chronic obstructive pulmonary disease, Physiology, Respiration

## Abstract

Decreasing exercise tolerance is one of the key features related to a poor prognosis in patients with chronic obstructive pulmonary disease (COPD). Cardiopulmonary exercise testing (CPET) is useful for evaluating exercise tolerance. The present study was performed to clarify the correlation between exercise tolerance and clinical parameters, focusing especially on the cross-sectional area (CSA) of skeletal muscle. The present study investigated 69 patients with COPD who underwent CPET. The correlations between oxygen uptake (${{\dot{\text{V}} \text{O}}}_{2}$) at peak exercise and clinical parameters of COPD, including skeletal muscle area measured using single-section axial computed tomography (CT), were evaluated. The COPD assessment test score (ρ = − 0.35, p = 0.02) was weakly correlated with ${{\dot{\text{V}} \text{O}}}_{2}$ at peak exercise. In addition, forced expiratory volume in one second (FEV_1_) (ρ = 0.39, p = 0.0009), FEV_1_/forced vital capacity (ρ = 0.33, p = 0.006), and the CSA of the pectoralis muscles (PMs) (ρ = 0.36, p = 0.007) and erector spinae muscles (ECMs) (ρ = 0.39, p = 0.003) were correlated with ${{\dot{\text{V}} \text{O}}}_{2}$ at peak exercise. Multivariate analysis adjusted by age and FEV_1_ indicated that PM_CSA_ was weakly correlated after adjustment (β value [95% confidence interval] 0.175 [0.03–0.319], p = 0.02). In addition, ECM_CSA_ tended to be correlated, but not significantly after adjustment (0.192 [− 0.001–0.385] p = 0.052). The COPD assessment test, FEV_1_, FEV_1_/FVC, PM_CSA_, and ECM_CSA_ were significantly correlated with ${{\dot{\text{V}} \text{O}}}_{2}$ at peak exercise.

## Introduction

Chronic pulmonary obstructive disease (COPD) is a common respiratory disease, with a reported global prevalence of 251 million cases^1^, and it is considered a life-threatening disease with decreasing pulmonary function and airflow limitation^2^.

Recently, factors related to a poor prognosis of COPD patients, including mortality and exacerbations, are becoming understood as evidence increases. For example, low-level physical activity, percent predicted forced expiratory volume in one second (%FEV_1_), 6-min walk distance, body mass index (BMI), and a high frequency of exacerbations are significantly associated with mortality in COPD patients^3,4^. We and others have also reported that low-level pulmonary function, exercise tolerance (including 6-min walk distance and exercise-induced desaturation), and BMI are correlated with a high frequency of exacerbations^5–8^, indicating that evaluations of exercise tolerance and body composition, in addition to pulmonary function, are important for predicting the clinical course of COPD.

Decreasing exercise tolerance, normally measured by the 6-min walk test or cardiopulmonary exercise testing (CPET), is one of the important clinical features related to a poor prognosis in COPD patients^3,9,10^, and with CPET one can evaluate exercise tolerance with exertional ventilatory parameters precisely and safely^11,12^. For example, oxygen uptake (${{\dot{\text{V}} \text{O}}}_{2}$) at peak exercise, which represents exercise tolerance, is significantly correlated with FEV_1_ and %FEV_1_ reflecting the severity of COPD^13,14^. Notably, with CPET, one can detect physical problems including cardiac dysfunction and functional skeletal muscle disorders during the test, which contributes to rapid initiation of treatment^15^.

Weight loss is a common systemic characteristic of patients with COPD^16^, and skeletal muscle loss has greater impact on the severity of COPD than decreased BMI^17^. Radiological analysis of skeletal muscles on computed tomography (CT) is a useful procedure for quantitation without onerous physical intervention^18,19^, and the cross-sectional area (CSA) of skeletal muscle on single-slice axial CT is significantly correlated with a poor prognosis in COPD patients^20,21^. In addition, the CSA of the erector spinae muscles (ECMs), which are anti-gravity muscles, but not of the pectoralis muscles (PMs), is significantly associated with mortality in Japanese patients with COPD^21^. Obviously, skeletal muscles are important for exercise tolerance, but the impact of exertional ventilatory parameters on CPET compared to clinical parameters in patients with COPD is not fully understood.

The aim of the present study was to identify the correlations between exertional ventilatory parameters, especially ${{\dot{\text{V}} \text{O}}}_{2}$ at peak exercise, and clinical parameters of COPD including skeletal muscle area. Our hypothesis was that skeletal muscle areas are correlated with ${{\dot{\text{V}} \text{O}}}_{2}$ at peak exercise, and the correlation coefficient of ECM_CSA_ is higher than that of PM_CSA_.

## Results

### Parameters of cardiopulmonary exercise testing

In the present study, 69 COPD patients (66 males, 3 females) who underwent CPET were enrolled. The clinical baseline characteristics of the COPD patients are shown in Table 1. ${{\dot{\text{V}} \text{O}}}_{2}$, which is a marker that reflects exercise tolerance^22^, was 295.6 ml/min at rest and 926.0 ml/min at peak exercise. Body weight-adjusted ${{\dot{\text{V}} \text{O}}}_{2}$ was 5.3 ml/min/kg at rest and 16.2 ml/min/kg at peak exercise. V_T_ and V_E_ were 773.2 ml and 12.9 l/min at rest and 1245.7 ml and 36.6 l/min at peak exercise, respectively. ${{\dot{\text{V}}}}$˙_E_/${{\dot{\text{V}}}}$_CO2_, which reflects pulmonary clearance of CO_2_^22^, was 49.3 at rest and 41.1 at peak exercise. V_D_/V_T_, which reflects the efficacy of pulmonary gas exchange, was 0.28 at rest and 0.26 at peak exercise. The respiratory rate was 17.7 breaths/min at rest and 30.5 breaths/min at peak exercise (Table 2).

**Table 1:** Demographics of the study participants (n = 69).

Clinical parameters	
Age (years)	71.1 ± 9.0
Gender (male/female)	66/3
BMI (kg/m^2^)	21.4 ± 3.8
Smoking history (pack-year)	67.6 ± 33.0
GOLD stage (I/II/III/IV, n)	12/27/25/5
mMRC dyspnea scale (0/1/2/3/4, n)	5/18/27/16/3
COPD assessment test (n = 41)	16.8 ± 7.6
6-min walk distance (n = 48) (m)	386.1 ± 115.8
**Medications**	
No respiratory medication, n (%)	5 (7.2%)
LAMA or LABA alone, n (%)	17 (24.6%)
LABA-LAMA combo, n (%)	19 (27.5%)
ICS-LABA combo, n (%)	9 (13.0%)
Triple combo, n (%)	19 (27.5%)
**Pulmonary function**	
%VC (%)	100.4 ± 18.4
%FVC (%)	95.2 ± 17.5
FEV_1_ (L)	1.35 ± 0.59
FEV_1_/FVC (%)	43.8 ± 13.4
%FEV_1_ (%)	60.2 ± 24.2
DLco (%)	65.9 ± 24.3
**Evaluation of skeletal muscle on CT (n = 56)**	
PM_CSA_ (cm^2^)	25.9 ± 7.9
ECM_CSA_ (cm^2^)	27.8 ± 6.2

*BMI* body mass index, *GOLD* global initiative for chronic obstructive lung disease, *mMRC* modified medical research council, *COPD* chronic obstructive pulmonary disease, *LAMA* long-acting muscarinic antagonist, *LABA* long acting β_2_ adrenergic agonist, *ICS* inhaled corticosteroid, *VC* vital capacity, *FVC* forced vital capacity, *FEV*_*1*_ forced expiratory volume in 1 s, *DLco* diffusing capacity of lung for carbon monoxide, *PM*_*CSA*_ cross-sectional area of pectoralis muscles, *ECM*_*CSA*_ cross-sectional area of erector spinae muscles. Data are presented as mean ± standard deviation.

**Table 2:** Results of cardiopulmonary exercise testing at rest and at peak exercise (n = 69).

	At rest	At peak exercise
**Incremental load testing**		
${{\dot{\text{V}} \text{O}}}_{2}$ (ml/min)	295.6 ± 68.2	926.0 ± 338.4
${{\dot{\text{V}} \text{O}}}_{2}$ (ml/min/kg)	5.3 ± 1.2	16.2 ± 4.7
V_T_ (ml)	773.2 ± 204.5	1245.7 ± 362.6
V_E_ (L/min)	12.9 ± 2.6	36.6 ± 10.9
${{\dot{\text{V}}}}$_E_/${{\dot{\text{V}}}}$_CO2_	49.3 ± 8.9	41.1 ± 8.4
V_D_/V_T_	0.28 ± 0.07	0.26 ± 0.07
Breathing frequency (times/min)	17.7 ± 4.2	30.5 ± 8.1

${{\dot{V}O}}_{2}$ oxygen uptake, *V*_*T*_ tidal volume, *V*_*E*_ minute ventilation, ${{\dot{{V}}}}$_*E*_/${{\dot{{V}}}}$_*CO2*_ ventilatory equivalent for carbon dioxide, *V*_*D*_*/V*_*T*_ dead space to tidal volume ratio. Data are presented as mean ± standard deviation.

### Correlations between ${{\dot{\text{V}} \text{O}}}_{2}$ (ml/min/kg) at peak exercise and other parameters on CPET and the 6-min walk distance

Because ${{\dot{\text{V}} \text{O}}}_{2}$ (ml/min) is affected by body weight differences, ${{\dot{\text{V}} \text{O}}}_{2}$ adjusted by body weight (ml/min/kg) at peak exercise is considered a precise marker for exercise tolerance^22^. Therefore, the evaluation focused on that and its correlations with other CPET parameters and the 6-min walk distance. ${{\dot{\text{V}} \text{O}}}_{2}$ at peak exercise was significantly correlated with ${{\dot{\text{V}}}}$_E_/${{\dot{\text{V}}}}$_CO2_ at rest (ρ = − 0.46, p < 0.0001) and at peak exercise (ρ = − 0.45, p < 0.0001), V_D_/V_T_ at rest (ρ = − 0.36, p = 0.002) and at peak exercise (ρ = − 0.53, p < 0.0001), respiratory rate at rest (ρ = − 0.35, p = 0.003) and at peak exercise (ρ = − 0.33, p = 0.006), and the 6-min walk distance (ρ = 0.74, p < 0.0001) (Table 3, Supplementary Fig. S2a online). These data showed that ${{\dot{\text{V}} \text{O}}}_{2}$ (ml/min/kg) at peak exercise reflected exercise tolerance in COPD patients.

**Table 3:** Correlation coefficients between ${{\dot{\text{V}} \text{O}}}_{2}$ at peak exercise and other CPET parameters and the 6-min walk distance.

	${{\dot{\text{V}}}}$_O2_ (ml/min/kg) at peak exercise	
	ρ	p value
${{\dot{\text{V}}}}$_E_/${{\dot{\text{V}}}}$_CO2_ at rest	− 0.46	< 0.0001
${{\dot{\text{V}}}}$_E_/${{\dot{\text{V}}}}$_CO2_ at peak exercise	− 0.45	< 0.0001
V_D_/V_T_ at rest	− 0.36	0.002
V_D_/V_T_ at peak exercise	− 0.53	< 0.0001
Breathing frequency at rest	− 0.35	0.003
Breathing frequency at peak exercise	− 0.33	0.006
6-min walk distance (n = 48)	0.74	< 0.0001

${{{\dot{V}O}}_{2}}$ oxygen uptake, *V*_*T*_ tidal volume, *V*_*E*_ minute ventilation, ${{\dot{{V}}}}$_*E*_/${{\dot{{V}}}}$_*CO2*_ ventilatory equivalent for carbon dioxide, *V*_*D*_*/V*_*T*_ dead space to tidal volume ratio.

### Correlations between ${{\dot{\text{V}} \text{O}}}_{2}$ (ml/min/kg) at peak exercise and clinical parameters of COPD including skeletal muscle area

**Figure 1:**
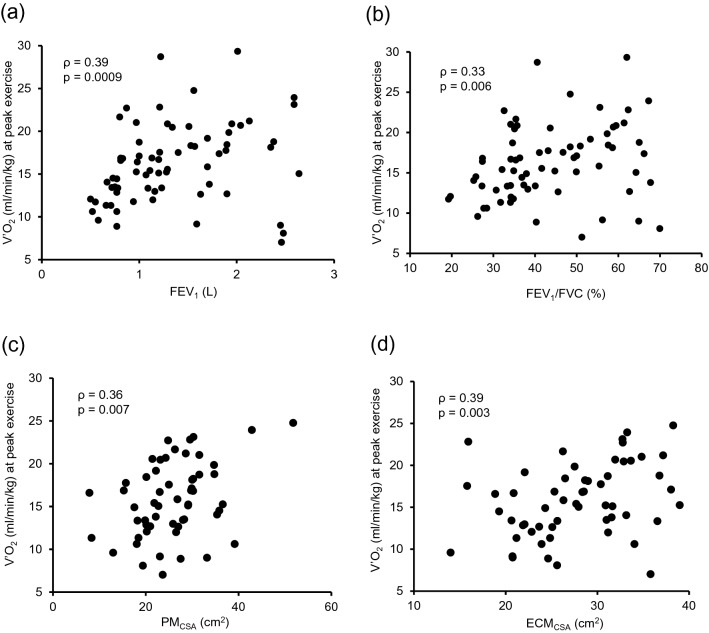
Correlations between ${{\dot{\text{V}} \text{O}}}_{2}$ at peak exercise and clinical parameters of COPD. Correlations between ${{\dot{\text{V}} \text{O}}}_{2}$ at peak exercise and (**a**) FEV_1_, (**b**) FEV_1_/FEV, (**c**) PM_CSA_, and (**d**) ECM_CSA_. ${{\dot{V}O}}_{2}$ oxygen uptake, *COPD* chronic obstructive pulmonary disease, *FEV*_*1*_ forced expiratory volume in 1 s, *FVC* forced vital capacity, *PM*_*CSA*_ cross-sectional area of the pectoralis muscles, *ECM*_*CSA*_ cross-sectional area of the erector spinae muscles.

**Figure 2:**
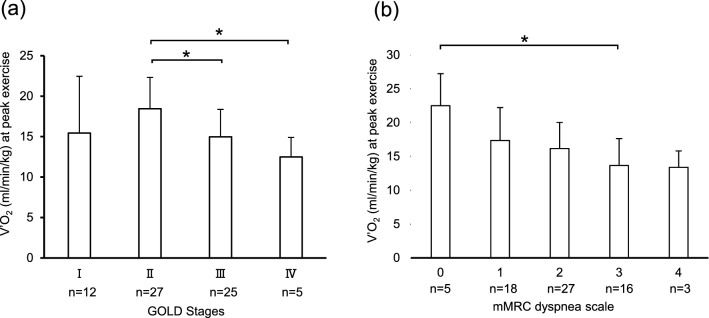
Results of ${{\dot{\text{V}} \text{O}}}_{2}$ by (**a**) GOLD stage and (**b**) mMRC dyspnea scale score. *p < 0.05. ${{\dot{V}O}}_{2}$ oxygen uptake, *GOLD* Global Initiative for Chronic Obstructive Lung Disease, *mMRC* modified Medical Research Council.

To clarify the factors correlated with exercise tolerance as reflected by ${{\dot{\text{V}} \text{O}}}_{2}$ (ml/min/kg) at peak exercise, correlation analysis between ${{\dot{\text{V}} \text{O}}}_{2}$ (ml/min/kg) at peak exercise and clinical parameters of COPD including skeletal muscle area was performed. Age, BMI, %VC, %FVC, %FEV_1_, and diffusing capacity of the lung for carbon monoxide (DLco) were not significantly correlated with ${{\dot{\text{V}} \text{O}}}_{2}$ at peak exercise. The COPD assessment test score (ρ = − 0.35, p = 0.02, Supplementary Fig. S2b online) was weakly correlated with ${{\dot{\text{V}} \text{O}}}_{2}$ at peak exercise. FEV_1_ (ρ = 0.39, p = 0.0009, Fig. 1a), FEV_1_/FVC (ρ = 0.33, p = 0.006, Fig. 1b), PM_CSA_ (ρ = 0.36, p = 0.007, Fig. 1c), and ECM_CSA_ (ρ = 0.39, p = 0.003, Fig. 1d) were significantly correlated with ${{\dot{\text{V}} \text{O}}}_{2}$ at peak exercise (Table 4). Examining the difference in ${{\dot{\text{V}} \text{O}}}_{2}$ at peak exercise by COPD stage, COPD stage III and IV patients had significantly lower levels of ${{\dot{\text{V}} \text{O}}}_{2}$ at peak exercise than stage II patients (Fig. 2a). In addition, examining the difference in ${{\dot{\text{V}} \text{O}}}_{2}$ at peak exercise by the mMRC dyspnea scale score, patients with an mMRC scale score of 3 had a significantly lower ${{\dot{\text{V}} \text{O}}}_{2}$ at peak exercise than those with an mMRC scale score of 0 (Fig. 2b). For other parameters on CPET, ${{\dot{\text{V}}}}$_E_/${{\dot{\text{V}}}}$_CO2_ at peak exercise was significantly correlated with BMI (ρ = − 0.33, p = 0.007), the COPD assessment test score (ρ = 0.58, p < 0.0001), DLco (ρ = − 0.42, p = 0.001), PM_CSA_ (ρ = − 0.32, p = 0.02), and ECM_CSA_ (ρ = − 0.34, p = 0.01). In addition, V_D_/V_T_ at peak exercise was significantly correlated with age (ρ = 0.34, p = 0.005), BMI (ρ = − 0.28, p = 0.02), the COPD assessment test score (ρ = 0.41, p = 0.009), %VC (ρ = − 0.28, p = 0.02), FEV_1_ (ρ = -0.42, p = 0.004), FEV_1_/FVC (ρ = − 0.36, p = 0.003), %FEV_1_ (ρ = − 0.27, p = 0.03), PM_CSA_ (ρ = − 0.35, p = 0.008), and ECM_CSA_ (ρ = − 0.38, p = 0.004) (Supplementary Table S1 online).

**Table 4:** Correlation coefficients between ${{\dot{\text{V}} \text{O}}}_{2}$ at peak exercise and clinical parameters of COPD including skeletal muscle area.

	${{\dot{\text{V}} \text{O}}}_{2}$ (ml/min/kg) at peak exercise	
	ρ	p value
Age (years)	− 0.22	0.08
BMI (kg/m^2^)	0.08	0.54
COPD assessment test	− 0.35	0.02
%VC (%)	0.19	0.11
%FVC (%)	0.16	0.2
FEV_1_ (L)	0.39	0.0009
FEV_1_/FVC (%)	0.33	0.006
%FEV_1_ (%)	0.24	0.05
DLco (%)	0.26	0.05
PM_CSA_ (cm^2^)	0.36	0.007
ECM_CSA_ (cm^2^)	0.39	0.003

*CPET* Cardiopulmonary exercise testing, ${{\dot{V}O}}_{2}$ oxygen uptake, BMI; body mass index, *COPD* chronic obstructive pulmonary disease, *VC* vital capacity, *FVC* forced vital capacity, *FEV*_*1*_ forced expiratory volume in 1 s, *DLco* diffusing capacity of lung for carbon monoxide, *PM*_*CSA*_ cross-sectional area of pectoralis muscles, *ECM*_*CSA*_ cross-sectional area of erector spinae muscles.

### Multivariate analysis of the correlation between ${{\dot{\text{V}} \text{O}}}_{2}$ at peak exercise and predictive variables including age, FEV_1_, and skeletal muscle areas

To evaluate the impact of skeletal muscle areas on exercise tolerance, multivariate analysis was performed using variables of age, FEV_1_ and skeletal muscle areas. PM_CSA_ (β value [95% confidence interval] 0.175 [0.03–0.319], p = 0.02) was weakly correlated after adjustment (Table 5). In addition, ECM_CSA_ (0.192 [− 0.001–0.385] p = 0.052) tended to be correlated, but not significantly after adjustment (Table 6).

**Table 5:** Multivariate analysis of correlations between ${{\dot{\text{V}} \text{O}}}_{2}$ at peak exercise and age, FEV_1_, and PM_CSA_ as predictive variables.

	Multivariate analysis		
	β	95% CI	p value
Age (years)	− 0.059	− 0.185–0.068	0.36
FEV_1_ (L)	0.826	− 1.01–2.662	0.37
PM_CSA_ (cm^2^)	0.175	0.03–0.319	0.02

*FEV*_*1*_ forced expiratory volume in 1 s, *ECM*_*CSA*_ cross-sectional area of erector spinae muscles, *β* standardized β value, *CI* confidence interval.

**Table 6:** Multivariate analysis of correlations between ${{\dot{\text{V}} \text{O}}}_{2}$ at peak exercise and age, FEV_1_, and ECM_CSA_ as predictive variables.

	Multivariate analysis		
	β	95% CI	p value
Age (years)	− 0.058	− 0.187–0.072	0.37
FEV_1_ (L)	0.785	− 1.099–2.668	0.41
ECM_CSA_ (cm^2^)	0.192	− 0.001–0.385	0.052

*FEV*_*1*_ forced expiratory volume in 1 s, *ECM*_*CSA*_ cross-sectional area of erector spinae muscles, *β* standardized β value, *CI* confidence interval.

## Discussion

In the present cross-sectional study, correlations between exercise tolerance indicated by ${{\dot{\text{V}} \text{O}}}_{2}$ at peak exercise and clinical parameters including skeletal muscle area were examined in Japanese patients with COPD. It was confirmed that ${{\dot{\text{V}} \text{O}}}_{2}$ at peak exercise was significantly correlated with 6-min walk distance and other CPET parameters, such as ${{\dot{\text{V}}}}$_E_/${{\dot{\text{V}}}}$_CO2_, V_D_/V_T_, and respiratory rate, which suggested that ${{\dot{\text{V}} \text{O}}}_{2}$ at peak exercise is a useful marker of exercise tolerance for COPD patients. The analysis of correlation coefficients showed that the COPD assessment test, FEV_1_, FEV_1_/FVC, PM_CSA_, and ECM_CSA_ were significantly correlated with ${{\dot{\text{V}} \text{O}}}_{2}$ at peak exercise, even though the correlations were weak. Additionally, the correlation coefficient between ${{\dot{\text{V}} \text{O}}}_{2}$ at peak exercise and ECM_CSA_ are comparable to that between ${{\dot{\text{V}} \text{O}}}_{2}$ at peak exercise and PM_CSA_.

Loss of exercise tolerance is an important and widely recognized clinical manifestation of COPD^15,22^. With respect to the mechanisms, exercise-induced dyspnea with dynamic pulmonary hyperinflation and desaturation of oxygen, which is a representative manifestation of COPD, contributes to a low threshold of exhaustion with the early appearance of anaerobic metabolites in skeletal muscles during exercise^22^. Thus, ${{\dot{\text{V}} \text{O}}}_{2}$ at peak exercise on CPET, which is determined by cellular O_2_ demand and the maximal rate of O_2_ transport, is considered a useful marker of exercise tolerance in COPD patients^15^. Diaz et al. analyzed 52 patients with mild to severe COPD, and air-flow limitation, which reflects the presence of dynamic hyperinflation, was found to be significantly associated with ${{\dot{\text{V}} \text{O}}}_{2}$ at peak exercise^12^. Moreover, Kagawa et al. analyzed 294 patients with COPD who underwent CPET, and they found that decreased FEV_1_ was associated with a low ${{\dot{\text{V}} \text{O}}}_{2}$ at peak exercise^13^. These reports showed that limitation of exercise tolerance predicted by decreased ${{\dot{\text{V}} \text{O}}}_{2}$ at peak exercise is an important phenotype of COPD, as shown in the present study (Table 4, Fig. 1a,b). The severity of COPD predicted by %FEV_1_ is also related to the decrease of exercise tolerance, and Yamamoto et al. reported that ${{\dot{\text{V}} \text{O}}}_{2}$ at peak exercise was significantly higher in COPD patients in GOLD stages I and II than in those in GOLD stages III and IV^14^. The current results also showed that the level of ${{\dot{\text{V}} \text{O}}}_{2}$ at peak exercise tended to be decreased depending on the GOLD stage, except for stage I (Fig. 2a), although the correlation between ${{\dot{\text{V}} \text{O}}}_{2}$ at peak exercise and %FEV_1_ was weak (Table 4). Notably, ${{\dot{\text{V}} \text{O}}}_{2}$ at peak exercise in GOLD stage II patients was higher than that in GOLD stage I patients, as shown in Fig. 2a, although the difference was not significant. As indicated in Fig. 1a, the level of ${{\dot{\text{V}} \text{O}}}_{2}$ at peak exercise has various values in patients who showed a high FEV_1_, which might indicate that exercise tolerance in the early stage of COPD involves factors except for airway limitation such as skeletal muscle mass.

Loss of skeletal muscles with bodyweight reduction, called sarcopenia, is also an important characteristic of COPD patients^20,23,24^. Reduction of fat-free mass containing skeletal muscle is associated with mortality in patients with COPD^25^. In addition, a previous report showed that COPD patients with decreased skeletal muscles, calculated by bioelectrical impedance analysis, walked a significantly shorter distance on the incremental shuttle walk test, which is another index of exercise tolerance, than those with preserved skeletal muscles^26^. With respect to the mechanisms, loss of skeletal muscles causes increased O_2_ demand as exercise intensity increases and earlier reaching of the anaerobic threshold with metabolic acidosis and increased lactate, which limits exercise tolerance in patients with COPD^27,28^. The present study showed that skeletal muscle area, including PM_CSA_ and ECM_CSA_, was significantly correlated with ${{\dot{\text{V}} \text{O}}}_{2}$ at peak exercise, which is consistent with these data (Table 4, Fig. 1c,d).

Notably, other gas exchange parameters on CPET such as ${{\dot{\text{V}}}}$_E_/${{\dot{\text{V}}}}$_CO2_ and V_D_/V_T_ at peak exercise were associated with the clinical data of COPD, including skeletal muscle area (Supplementary Table S1 online). These parameters were reported to be significantly higher in patients with COPD than in healthy individuals^29^, and ${{\dot{\text{V}}}}$_E_/${{\dot{\text{V}}}}$_CO2_, which reflects decreased pulmonary clearance of CO_2_ during exercise, was correlated with BMI, %FEV_1_, and DLco, in addition to skeletal muscle area. Moreover, V_D_/V_T_, which reflects worse pulmonary gas exchange efficacy, was correlated with age, BMI, %VC, FEV_1_, FEV_1_/FVC, and %FEV_1_, in addition to skeletal muscle area. Interestingly, the COPD assessment test score was strongly correlated with these parameters, suggesting that ${{\dot{\text{V}}}}$_E_/${{\dot{\text{V}}}}$_CO2_ and V_D_/V_T_ might reflect COPD-related symptoms (Supplementary Table S1 online).

The present study has several limitations. First, correlations with physical activity were not evaluated. Second, correlations were evaluated using clinical parameters of COPD and skeletal muscle area, which acted as confounding factors. Third, study participants were selected by physicians’ suggestions and patients’ acceptance, which might have caused selection bias. Fourth, it is unclear that the current results for the correlation between exercise tolerance and skeletal muscle area is specific for patients with COPD, because healthy controls were not included. Fifth, the present study involved patients at a single hospital with limited ethnic diversity and a small sample size. Additionally, the percentage of females was extremely low in the present study, consistent with the general population of COPD, which might affect generalizability. To confirm the validity of the present results, multicenter, prospective studies with a larger number of patients should be performed.

## Conclusions

The present cross-sectional study showed that in FEV_1_, FEV_1_/FVC, and skeletal muscle areas including PM_CSA_ and ECM_CSA_ are significantly correlated with exercise tolerance, even though the correlations are weak. These data suggest that pulmonary function and skeletal muscles contribute to exercise tolerance in patients with COPD.

## Methods

### Study design

The cross-sectional study was designed following the recommendations of the STROBE statement and approved by the ethics committee of Saga University Hospital (approval number: 2020-11-R-03, approval date: Jan 27, 2021) in accordance with the 1964 Declaration of Helsinki. Informed consent of the participants was obtained in the form of opt-out on the website. Those who rejected were excluded.

### Patients and setting

The medical records of 69 patients diagnosed with COPD who underwent CPET at the Saga University Hospital between 2009 and 2020 were included in the present study. All patients satisfied the definition criteria of the Global Initiative for Chronic Obstructive Lung Disease (GOLD). Briefly, patients were confirmed to have FEV_1_/FEV < 0.7 after using a bronchodilator, a smoking index > 10 pack years, and symptoms including chronic cough, sputum, and dyspnea. Patients with either a current or a previous diagnosis of asthma were excluded. For patient information, age at the time CPET was performed was used, and clinical parameters including BMI, modified Medical Research Council (mMRC) dyspnea scale, COPD assessment test, 6-min walk test, medication record, and pulmonary function at the time closest to when CPET was performed (within ± 3 months) were evaluated. Thus, 41 patients who underwent the COPD assessment test and 48 patients who underwent the 6-min walk test were analyzed. Medications were selected at each physician’s discretion. For handling of missing values, the participant data record was excluded for waves of data collection with missing values. The primary outcome was set as a significant correlation between ${{\dot{\text{V}} \text{O}}}_{2}$ at peak exercise and skeletal muscle areas including PM_CSA_ and ECM_CSA_. For sample size calculation, the correlation between ${{\dot{\text{V}} \text{O}}}_{2}$ at peak exercise and skeletal muscle area have not been assessed, to the best of our knowledge, which suggests that the accurate calculation was not feasible. However, previous studies reported that FEV_1_ was significantly correlated with ${{\dot{\text{V}} \text{O}}}_{2}$ at peak exercise ^14^ and skeletal muscle area^20^. We hypothesized a significant correlation between ${{\dot{\text{V}} \text{O}}}_{2}$ at peak exercise and skeletal muscle area as with FEV_1_ (r = 0.4) and performed test of no correlation with two-sided 0.05 of significant level and 0.8 of statistical power, which estimated a sample size of 47 patients. Thus, we considered the current sample size of 69 patients was sufficient to achieve this primary outcome.

### Cardiopulmonary exercise testing

A symptom-limited cycle ergometer (Strength Ergo 8, Mitsubishi Electric Engineering, Japan) was used for CPET. Each patient wore a mask, and breath was analyzed using a gas analyzer (Cpex-1, Inter Reha; Japan); ${{\dot{\text{V}} \text{O}}}_{2}$, expiratory tidal volume (V_T_), minute ventilation (V_E_), ventilatory equivalent for carbon dioxide (${{\dot{\text{V}}}}$_E_/${{\dot{\text{V}}}}$_CO2_), dead space to tidal volume ratio (V_D_/V_T_), and breathing frequency at rest and at peak exercise were evaluated. Oxygen saturation, blood pressure, and the electrocardiogram were measured during the test. In the exercise protocol, pre-exercise resting measurements were obtained within the steady state period for more than 3 min. Incremental testing was then started by increasing the load by 10 W per minute with a ramp-exercise protocol. The examination was continued until exhaustion or the predicted maximum heart rate or blood pressure was surpassed, and showing electrocardiographic changes such as ST segment depression of greater than 2 mm and a short run of premature ventricular contractions. Dyspnea intensity was evaluated by a 10-point modified Borg category-ratio scale at rest and every 1 min after initiation of the incremental load test. The data generated were measured breath-by-breath and as 30-s averages at rest and during exercise.

### CT scan acquisition and analysis

Chest CT for analysis of the pectoralis and erector spinae muscles that was performed most closely to the time of CPET (within ± 3 years) was also selected; the average time between CPET and chest CT was 198 days. Consequently, 56 patients were examined. For quantitative analysis, the CSAs of the pectoralis muscles (PM_CSA_) and the erector spinae muscles (ESM_CSA_) were evaluated referring to the previously described method^20,24,30^. Briefly, left and right areas of the PM_CSA_ identified by the superior aspect of the aortic arch and the ESM_CSA_ identified by the lower aspect of the 12th thoracic vertebrae on CT imaging reconstructed using the mediastinal setting were identified and shaded manually. Finally, the sum of the left and right muscle areas was examined. The measurements were performed by two pulmonary physicians independently referring to the representative images (Supplemental Fig. S1a,b online), and average values were used.

### Statistical analysis

For correlation analysis, Spearman’s rank correlation coefficients between exercise tolerance parameters such as ${{\dot{\text{V}} \text{O}}}_{2}$, ${{\dot{\text{V}}}}$_E_/${{\dot{\text{V}}}}$_CO2_, V_D_/V_T_, breathing frequency, and 6-min walk distance, and clinical parameters including age, BMI, COPD assessment test score, %VC, %FVC, FEV_1_, FEV_1_/FVC, %FEV_1_, DLco, PM_CSA_, and ECM_CSA_ were calculated to determine whether they were zero. Differences of ${{\dot{\text{V}} \text{O}}}_{2}$ at peak exercise depending on GOLD stages and the mMRC dyspnea scale were analyzed by the Steel–Dwass method. Multivariate analysis with linear regression analysis was performed for continuous variables, and β coefficient values were calculated. Quantitative data are presented as means ± standard deviation (SD); significance was considered a p value less than 0.05. Statistical analysis was performed with JMP Pro version 14.2.0 software (SAS Institute Inc., Cary, NC, USA).

## Supplementary Information

Supplementary Information 1.

Supplementary Figures.

## Data Availability

The datasets used and analyzed during the present study are available from the corresponding author on reasonable request.
