# Supplementary material for: Skeletal muscle is associated with exercise tolerance evaluated by cardiopulmonary exercise testing in Japanese patients with chronic obstructive pulmonary disease
Source: Sci Rep. 2021 Aug 5;11:15862. doi: 10.1038/s41598-021-95413-9 (PMC8342424; doi:10.1038/s41598-021-95413-9)
Supplement: Supplementary file 1 — Supplementary Information 1. [file 41598_2021_95413_MOESM1_ESM.docx]

**Supplementary Information**

**Title**

Skeletal muscle is associated with exercise tolerance evaluated by cardiopulmonary exercise testing in Japanese patients with chronic obstructive pulmonary disease

**Authors**

Hiroki Tashiro^1^, Koichiro Takahashi^1^, Masahide Tanaka^1^, Hironori Sadamatsu^1^, Yuki Kurihara^1^, Ryo Tajiri^2^, Ayako Takamori^2^, Hiroyuki Naotsuka^3^, Hiroki Imaizumi^3^, Shinya Kimura^1^, Naoko Sueoka-Aragane^1^

**Affiliations**

^1^ Division of Hematology, Respiratory Medicine and Oncology, Department of Internal Medicine, Faculty of Medicine, Saga University, Saga, Japan

^2^ Clinical Research Center, Saga University Hospital, Saga, Japan

^3^ Advanced Comprehensive Functional Recovery Center, Saga University Hospital, Saga, Japan

Figure S1. Representative computed tomographic images referred to measure the cross-sectional area of skeletal muscles. The erector spinae muscles (a) and the pectoralis muscles (b) are surrounded by green line.

Figure S2. Correlation between V̇_O2_ at peak exercise and (a) 6-minute walk distance, (b) the COPD assessment test

V̇O_2_: oxygen uptake

Table S1. Correlation coefficient between V̇_E_/V̇_CO2_, V_D_/V_T_ at peak exercise and clinical parameters of COPD

|  | V̇_E_/V̇_CO2_ at peak exercise | | V_D_/V_T_ at peak exercise | |
| --- | --- | --- | --- | --- |
|  | ρ | p value | ρ | p value |
| Age (years) | 0.06 | 0.6 | 0.34 | 0.005 |
| BMI (kg/m^2^) | -0.33 | 0.007 | -0.28 | 0.02 |
| COPD assessment test | 0.58 | <0.0001 | 0.41 | 0.009 |
| %VC (%) | 0.09 | 0.45 | -0.28 | 0.02 |
| %FVC (%) | 0.15 | 0.23 | -0.18 | 0.14 |
| FEV_1_ (L) | -0.05 | 0.71 | -0.42 | 0.004 |
| FEV_1_/FVC (%) | -0.09 | 0.46 | -0.36 | 0.003 |
| %FEV_1_ (%) | 0.08 | 0.5 | -0.27 | 0.03 |
| DLco (%) | -0.42 | 0.001 | -0.19 | 0.15 |
| PM_CSA_ (cm^2^) | -0.32 | 0.02 | -0.35 | 0.008 |
| ECM_CSA_ (cm^2^) | -0.34 | 0.01 | -0.38 | 0.004 |

V̇_E_/V̇_CO2_; ventilatory equivalent for carbon dioxide, V_D_/V_T_; dead space to tidal volume ratio, BMI; body mass index, GOLD; global initiative for chronic obstructive lung disease, mMRC; modified medical research council, COPD; chronic obstructive pulmonary disease, LAMA; long-acting muscarinic antagonist, LABA; long acting β_2_ adrenergic agonist, ICS; inhaled corticosteroid, VC; vital capacity, FVC; forced vital capacity, FEV_1_; forced expiratory volume in 1 second, DLco; diffusing capacity of lung for carbon monoxide, PM_CSA_; cross-sectional area of pectoralis muscles, ECM_CSA_ ; cross-sectional area of erector spinae muscles.
