# Supplementary material for: Skeletal muscle is associated with exercise tolerance evaluated by cardiopulmonary exercise testing in Japanese patients with chronic obstructive pulmonary disease
Source: Sci Rep. 2021 Aug 5;11:15862. doi: 10.1038/s41598-021-95413-9 (PMC8342424; doi:10.1038/s41598-021-95413-9)
Supplement: Supplementary file 2 — Supplementary Figures. [file 41598_2021_95413_MOESM2_ESM.pptx]

## Slide 1
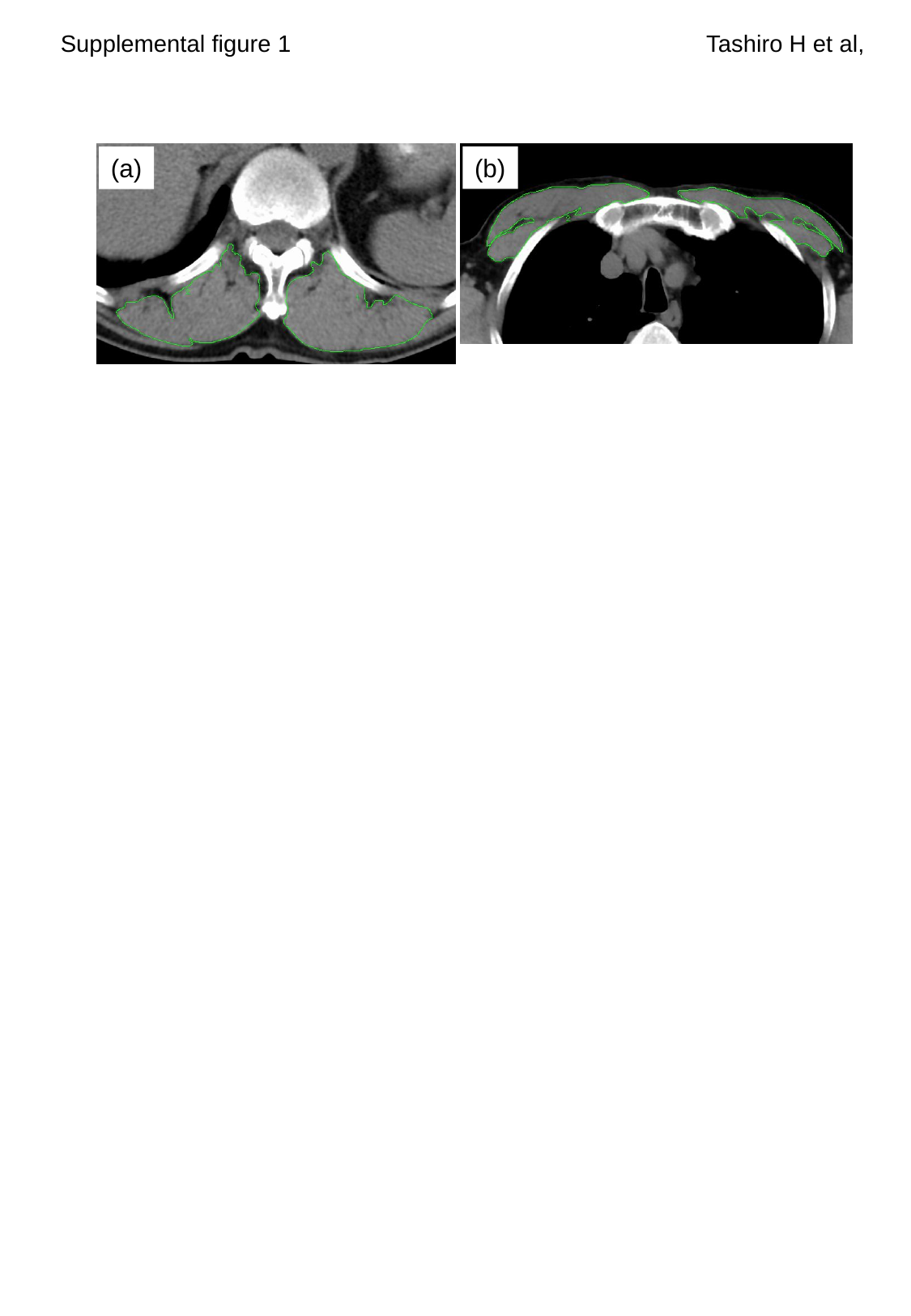

Supplemental figure 1
Tashiro H et al,
(b)
(a)

## Slide 2
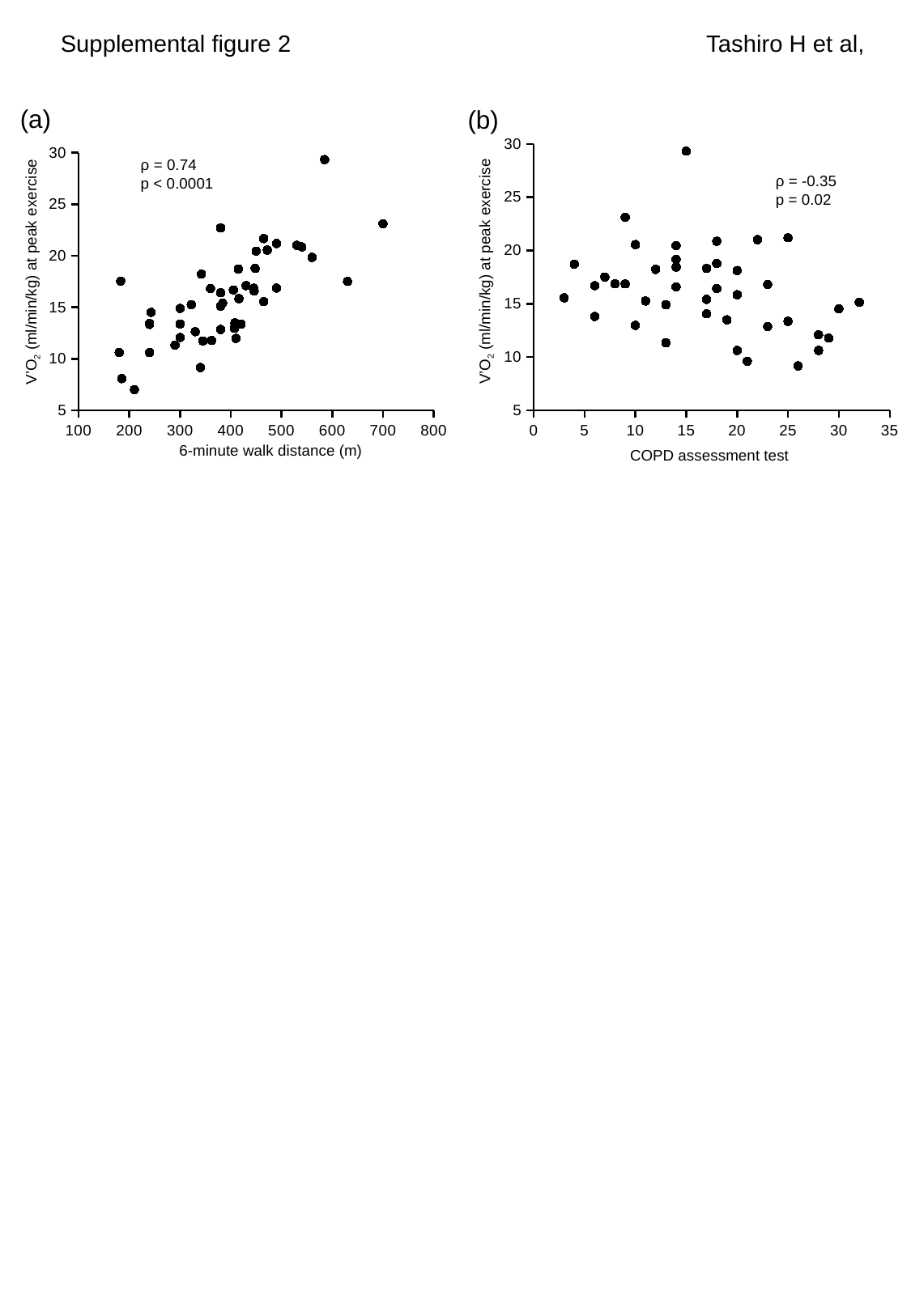

Supplemental figure 2
Tashiro H et al,
(a)
(b)
### Chart
| Category | 11.33479212 |
|---|---|
### Chart
| Category | |
|---|---|ρ = 0.74
p < 0.0001
ρ = -0.35
p = 0.02
V’O2 (ml/min/kg) at peak exercise
V’O2 (ml/min/kg) at peak exercise
6-minute walk distance (m)
COPD assessment test
